# New Forms of Scalers

**Published:** 1885-10

**Authors:** 


### NEW FORMS OF SCALERS.

We have received from Dr. A. W. Harlan, of Chicago, a pair of double curve, right and left scalers, that are beautifully adapted to the removal of deposits from the inside of the lower incisors, and from the proximal surfaces of the posterior teeth.

Dr. Harlan also sends copies of blank charts, showing the position and character of deposits upon the teeth of patients, which dentists are requested to fill up and return to him. It is well known that he has for some time made a special study of such cases, and practitioners may assist him, and themselves finally reap the rewards of his labors, if they will bestow a little time in registering their pyorrhœa cases, and afterwards transmit the charts to him. We presume that any one can obtain copies by addressing Dr. Harlan at Chicago.

The diseases caused by the deposits of calcareous matter upon teeth are now receiving a great deal of attention, as is evidenced by the two articles upon instruments adapted to its removal in the present number of this journal. Many dentists, feeling the need of scalers that were not to be found in the dental depots, have devised them originally, and the whole profession will benefit by their skill. At the late meeting of the American Dental Association, Dr. Allport, of Chicago, exhibited such a set, and they were greatly admired.

